# WNK4 is the major WNK positively regulating NCC in the mouse kidney

**DOI:** 10.1042/BSR20140047

**Published:** 2014-05-09

**Authors:** Daiei Takahashi, Takayasu Mori, Naohiro Nomura, Muhammad Zakir Hossain Khan, Yuya Araki, Moko Zeniya, Eisei Sohara, Tatemitsu Rai, Sei Sasaki, Shinichi Uchida

**Affiliations:** *Department of Nephrology, Graduate School of Medical and Dental Sciences, Tokyo Medical and Dental University, 1-5-45 Yushima, Bunkyo, Tokyo 113-8519, Japan

**Keywords:** angiotensin II, distal convoluted tubule, hypertension, kidney, Na–Cl co-transporter, with-no-lysine kinase (WNK), Akt, also called protein kinase B (PKB), AngII, angiotensin II, BAC, bacterial artificial chromosome, BP, blood pressure, DCT, distal convoluted tubule, ENaC, epithelial Na^+^ channel, ES, embryonic stem, KLHL3, Kelch-like family member 3, NCC, Na–Cl co-transporter, NKCC2, Na-K-Cl co-transporter isoform 2, OSR1, oxidative stress-responsive 1, PHAII, pseudohypoaldosteronism type II, RAA, renin–angiotensin–aldosterone, ROMK, renal outer medullary K^+^ channel, SPAK, Ste20-like proline/alanine-rich kinase, TG, transgenic, WNK, with-no-lysine kinase

## Abstract

By analysing the pathogenesis of a hereditary hypertensive disease, PHAII (pseudohypoaldosteronism type II), we previously discovered that WNK (with-no-lysine kinase)–OSR1/SPAK (oxidative stress-responsive 1/Ste20-like proline/alanine-rich kinase) cascade regulates NCC (Na–Cl co-transporter) in the DCT (distal convoluted tubules) of the kidney. However, the role of WNK4 in the regulation of NCC remains controversial. To address this, we generated and analysed WNK4^−/−^ mice. Although a moderate decrease in SPAK phosphorylation and a marked increase in WNK1 expression were evident in the kidneys of WNK4^−/−^ mice, the amount of phosphorylated and total NCC decreased to almost undetectable levels, indicating that WNK4 is the major WNK positively regulating NCC, and that WNK1 cannot compensate for WNK4 deficiency in the DCT. Insulin- and low-potassium diet-induced NCC phosphorylation were abolished in WNK4^−/−^ mice, establishing that both signals to NCC were mediated by WNK4. As shown previously, a high-salt diet decreases phosphorylated and total NCC in WNK4^+/+^ mice via AngII (angiotensin II) and aldosterone suppression. This was not ameliorated by WNK4 knock out, excluding the negative regulation of WNK4 on NCC postulated to be active in the absence of AngII stimulation. Thus, WNK4 is the major positive regulator of NCC in the kidneys.

## Abstract 2

The analyses of WNK4 (with-no-lysine kinase 4) knockout mice help to end a long-standing controversy about the role of WNK4 on NCC (Na–Cl co-transporter) regulations in the kidney. WNK4 is a strong positive regulator of NCC.

## INTRODUCTION

PHAII (pseudohypoaldosteronism type II) is a hereditary hypertensive disease characterized by hypokalaemia, metabolic acidosis and thiazide sensitivity [1]. In 2001, *WNK (with-no-lysine kinase*) *1* and *WNK*4 were identified as the genes responsible for PHAII [2]. The high thiazide sensitivity of PHAII suggested that activation of the NCC (Na–Cl co-transporter) in the DCT (distal convoluted tubules) of the kidneys was responsible for its pathogenesis. Therefore, several investigations on the regulation of NCC by WNKs were performed [3,4]. Initially, some of these studies reported that wild-type WNK4 negatively regulated NCC in *Xenopus* oocytes [5,6], which was reportedly not kinase activity-dependent function of WNK4 [7,8]. However, we generated and analysed PHAII model mice (*WNK*4*^D561A/+^*) carrying a PHAII-causing mutation in WNK4, and discovered a novel signalling cascade between WNK and NCC via OSR1 (oxidative stress-responsive 1) and SPAK (Ste20-like proline/alanine-rich kinase) in the kidney [9,10]. We reported that constitutive activation of this signalling cascade caused the molecular pathogenesis of PHAII induced by WNK4 mutation [10,11]. Because both WNK1 and WNK4 were demonstrated to positively influence OSR1 and SPAK [12], this kinase activity-dependent effect of WNK4 must be acting positively on NCC in the kidneys. Thus, two opposing methods of NCC regulation by WNK4 were postulated, and the identity of the major mechanism of NCC regulation in the kidneys *in vivo* remained unclear. Previously, Castañeda-Bueno et al. [13] reported the analysis of WNK4 knockout mice. However, this group focused on the role of WNK4 in AngII (angiotensin II)- or low-salt diet-induced activation of NCC, and did not completely investigate the controversial role of WNK4 in NCC regulation.

Mutations in two additional genes, KLHL3 (*Kelch-like family member 3*) and *Cullin-3*, were recently reported to cause PHAII [14,15]. We clarified that WNK4 is a substrate of the KLHL3–Cullin-3 E3 ligase complex, and that the impaired ubiquitination of WNK4 protein and, subsequent, increase of WNK4 protein abundance in DCT would represent a common molecular pathogenesis of PHAII caused by mutations in *WNK*4, *KLHL3* or *Cullin-3* [16]. In fact, we demonstrated that WNK4 protein is increased in *WNK*4*^D561A/+^* PHAII model mice [16]. These recent data strongly support a positive regulatory role for WNK4 in NCC regulation.

To obtain a definite conclusion regarding the physiological role of WNK4 in the kidneys, a more detailed examination of WNK4 knockout mice would be necessary. Previously, we generated hypomorphic WNK4 knockout mice, with the reduced function of WNK4, and obtained results suggesting that WNK4 acts positively on NCC in the kidneys [17]. However, a definite conclusion must be presented by analysing complete WNK4 knock out; to address this, we generated WNK4^−/−^ mice and examined them under various conditions. Because we had clarified that NCC is regulated by diets and some hormonal factors other than AngII, we first attempted to clarify whether NCC regulations other than that by AngII are WNK4-dependent. In addition, we investigated whether there was any evidence in the kidneys that supports a negative role of WNK4 in NCC regulation.

## MATERIALS AND METHODS

### Generation of WNK4 knockout mice

To generate WNK4^−/−^ mice, we prepared a BAC (bacterial artificial chromosome) clone bMQ42809 containing the mouse genomic *WNK*4 locus. The targeting vector was then transfected into J1-6 ES (embryonic stem) cells by electroporation, as previously described [18]. After selection with 150 μg/ml G418 and 2 μM ganciclovir, the targeted ES cell clones were selected by PCR. Chimeric male mice were bred with C57BL/6J females to produce heterozygous floxed (WNK4^flox/+^) mice, and the neo cassette was then deleted by crossing the WNK4^flox/+^ mice with Cre recombinase-expressing TG (transgenic) mice [19]. Genotyping of the mice was performed by PCR using the sense primer F (5′-ACAAAGGCGCTATTGAGTGC-3′) and the antisense primer R1 (5′-CGTCTGGGTCGGAAAGAAACT-3′) to detect the flanked exon 2. Further PCR analysis was performed using the sense primer F and the antisense primer R2 (5′-CAAGAAGAGCATGGGACATC-3′) to detect the upper loxP site.

Studies were performed on 12–16-week-old WNK4^+/+^, WNK4^+/−^ and WNK4^−/−^ littermates. The mice were raised under a 12-h day and night cycle, and were fed a normal rodent diet and plain drinking water. This experiment was approved by the Animal Care and Use Committee of the Tokyo Medical and Dental University, Tokyo, Japan.

### Blood measurements

Blood for electrolyte analysis was obtained as previously described [9]. Electrolyte levels were determined using an i-STAT® Portable Clinical Analyzer (Fuso Pharmaceutical Industries Ltd). Samples for plasma aldosterone measurement were obtained from the inferior vena cava under anaesthesia with pentobarbital. Plasma aldosterone levels were measured by SRL Clinical Laboratory Services.

### Immunoblotting and immunofluorescence

Extraction of kidney protein samples, semi-quantitative immunoblotting and immunofluorescence were performed as previously described [9]. For semi-quantitative immunoblotting, we used entire kidney samples without the nuclear fraction (600 ***g***) or the crude membrane fraction (17000 ***g***). The relative intensities of immunoblot bands were analysed and quantified using ImageJ (National Institutes of Health) software. The primary antibodies used in this study were as follows: rabbit anti-WNK1 (A301-516A; Bethyl Laboratories); anti-WNK4 [17]; rabbit anti-phosphorylated SPAK [20]; rabbit anti-SPAK (Cell Signaling Technology, Inc.); anti-phosphorylated OSR1/SPAK [17]; anti-OSR1 (M10; Abnova Corporation); rabbit anti-phosphorylated NCC (S71) [21]; guinea pig anti-NCC [22,23]; rabbit anti-ENaC (epithelial Na^+^ channel) α subunit (Chemicon International); rabbit anti-ENaC γ subunit (provided by M. Knepper, NIH) [24]; rabbit anti-ROMK (renal outer medullary K^+^ channel; provided by P. A. Welling and J. B. Wade, Maryland University) [25]; rabbit anti-actin (Cytoskeleton, Inc.); rabbit anti-phosphorylated NKCC2 (Na-K-Cl co-transporter isoform 2) [26]; guinea pig anti-NKCC2 (provided by K. Mutig, Department of Anatomy, Charité-Universitätsmedizin, Berlin) [27]; rabbit anti-phosphorylated serine/threonine protein kinase Akt [PKB (protein kinase B: also called Akt); Cell Signaling Technology, Inc.]; and rabbit anti-Akt (Santa Cruz Biotechnology, Inc.). ALP (alkaline phosphatase)-conjugated anti-IgG antibodies (Promega Corporation) were used as secondary antibodies and Western Blue® (Promega Corporation) was used to detect the signal.

### Blood pressure (BP) measurement

We measured the systolic BP using implantable radiotelemetric devices. The equipment for conscious, freely moving laboratory animals was purchased from Data Sciences International, which included an implantable transmitter (model TA11PA-C10), a receiver (model RPC-1), a data-processing device (Data Exchange Matrix) and an APR-1 (ambient pressure reference monitor). All data were computed using an analysis program (Dataquest ART4.31).

### Statistical analysis

Comparisons between the two groups were performed using unpaired *t*-tests. One-way ANOVA with Tukey's *post hoc* test was used to evaluate statistical significance in the comparisons between multiple groups. *P*-values <0.05 were considered statistically significant. Data are presented as the means±S.E.M.

## RESULTS

### Generation of WNK4*^−^*^/^*^−^* mice

**Figure 1:**
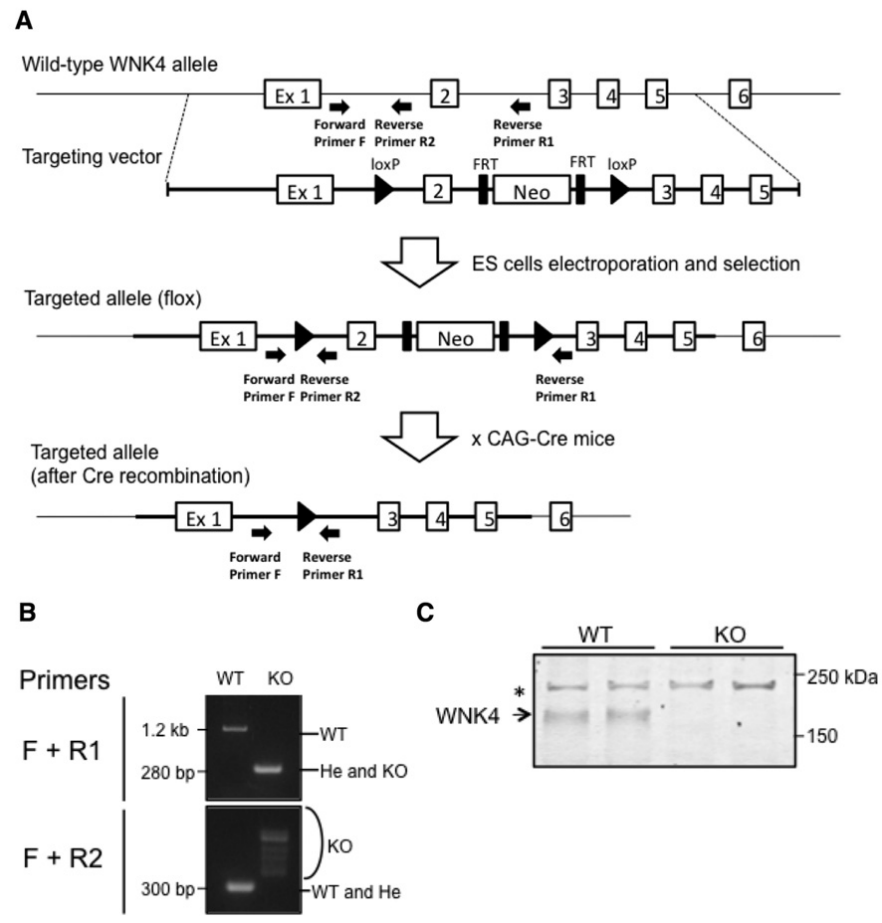
Generation of WNK4*^−^*^/^*^−^* mice (**A**) Targeting strategy to generate WNK4 knockout mice. This diagram shows the targeting construct, the wild-type WNK4 locus, and the targeted locus before and after Cre recombination. Exon 2 was flanked by two *loxP* sites. (**B**) Genotyping PCR after Cre recombination using primer sets flanking exon 2 (Primer F and R1) and flanking the upper *loxP* site (Primer F and R2). In the upper panel, the 280-bp band represents the mutant allele containing the remaining loxP site, whereas the 1.2-kb band represents the wild-type allele. In the lower panel, the 300-bp band represents the wild-type allele, whereas the smeared band represents the homozygous knockout alleles. (**C**) Immunoblotting of WNK4 from the kidneys of WNK^+/+^ and WNK4*^−^*^/^*^−^* mice. We used 40 μg of total protein per lane. The asterisk indicates non-specific bands [22].

To generate WNK4*^−^*^/^*^−^* mice, we designed a targeting vector to delete a single exon (exon 2) of the *WNK*4 (Figure 1A). Homologous recombination was confirmed by PCR in ES cell lines, which were used to generate WNK4^flox/+^ mice. Next we crossed WNK4^flox/+^ mice with Cre recombinase-expressing TG mice. The Cre-mediated excision of exon 2 in heterozygous (WNK4^+/^*^−^*) mice was verified by PCR. Homozygous knockout (WNK4*^−^*^/^*^−^*) mice were successfully generated by crossing WNK4^+/^*^−^* mice (Figure 1B). Heterozygous and homozygous WNK4 knockout mice exhibited no gross anatomic or behavioural abnormalities, and presented a normal birth rate. Absence of WNK4 protein in WNK4*^−^*^/^*^−^* mice was confirmed by immunoblotting of the kidney proteins (Figure 1C).

### General characteristics of WNK4*^−^*^/^*^−^* mice

First, we compared venous blood chemistry between WNK4*^−^*^/^*^−^* mice and their wild-type littermates fed with a normal diet (Table 1). WNK4*^−^*^/^*^−^* mice exhibited higher plasma pH and lower plasma Na^+^ and Cl^−^ than the wild-type littermates. There was no significant difference in plasma K^+^ between the two groups; this was in contrast to the findings of Castañeda-Bueno et al. [13].

**Table 1:** Blood biochemistry of WNK4^+/+^ and WNK4^−/−^ mice Values are the means±S.E.M.

	WNK4 WT (*n*=16)	WNK4 KO (*n*=17)	*P*
Na (mM)	146.3±0.4	144.4±0.3	<0.05
K (mM)	4.58±0.15	4.59±0.15	0.958
Cl (mM)	113.1±0.6	109.2±0.8	<0.01
Glu (mg/dl)	206.9±8.1	203.4±7.3	0.737
pH	7.237±0.01	7.298±0.01	<0.001
PCO_2_ (mmHg)	57.0±1.8	52.8±1.6	0.084
HCO_3_^−^ (mM)	24.2±0.5	25.9±0.8	0.073
BE	−3.3±0.5	−0.6±0.9	<0.05
Hb (g/dl)	15.1±0.2	15.6±0.2	0.092

**Figure 2:**
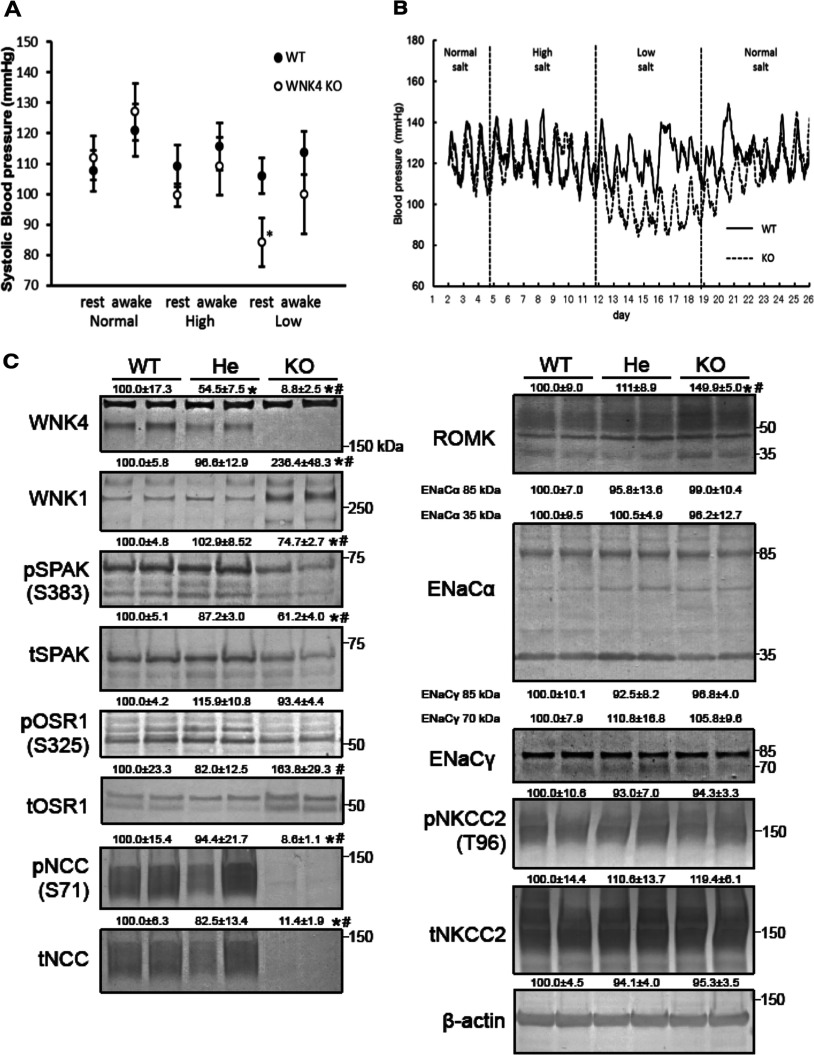
General characteristics of WNK4*^−^*^/^*^−^* mice (**A**) Radiotelemetric analyses of BP in WNK4^+/+^ and WNK4*^−^*^/^*^−^* male mice receiving diets with differing salt contents. Systolic BP in WNK4^+/+^ mice remained stable in response to diets with differing salt contents. In contrast, WNK4*^−^*^/^*^−^* mice exhibited a significantly lower BP when fed a low-salt diet (*P<0.05). (**B**) Representative radiotelemetric measurements of BP. Although WNK4^+/+^ mice were unaffected by diets with differing salt contents, WNK4*^−^*^/^*^−^* mice exhibited lower BP when fed a low-salt diet. BP returned to normal when dietary salt reverted to normal levels. (**C**) Representative immunoblotting analyses of kidney proteins in WNK4^+/+^ (WT), WNK4^+/^*^−^* (He), and WNK4*^−^*^/^*^−^* (KO) mice fed a normal diet. The numbers reflect the results of semi-quantification by densitometry (*P<0.05 versus WNK4^+/+^ mice, #*P*<0.05 versus WNK4^+/^*^−^* mice; *n*=5, expressed as percentages, means±S.E.M.). We used 40 μg of total protein per lane. WNK4 in WNK4^+/^*^−^* mice was about half that in wild-type mice, and was absent in WNK4*^−^*^/^*^−^* mice. WNK1 increased in WNK4*^−^*^/^*^−^* mice. Total and phosphorylated SPAK expression decreased in WNK4*^−^*^/^*^−^* mice. Total OSR1 expression increased in WNK4*^−^*^/^*^−^* mice. NCC expression did not decrease in WNK4^+/^*^−^* mice, but NCC was almost completely absent in WNK4*^−^*^/^*^−^* mice. ROMK expression increased in WNK4*^−^*^/^*^−^* mice.

Next, we analysed the effect of dietary salt intake on the BP in WNK4*^−^*^/^*^−^* mice. We used radiotelemetric devices to measure the BP of WNK4*^−^*^/^*^−^* mice and their wild-type littermates at different dietary sodium levels. Male mice were fed normal [0.9% NaCl (w/w)], high- [4% NaCl (w/w)] or low- [0.01% (w/w)] sodium diets for 7 days. As shown in Figure 2, no significant difference was evident while they received the normal or high-sodium diets. However, on the low-salt diet, the systolic BP of WNK4*^−^*^/^*^−^* mice was significantly lower than that of WNK4^+/+^ mice during the resting period (day time; 105.9±5.84 versus 84.1±8.07 mm Hg; *n*=3; *P*<0.05; Figure 2A).

### Expression of proteins associated with the WNK–OSR1/SPAK–NCC phosphorylation cascade

We investigated the expression of proteins associated with the WNK–OSR1/SPAK–NCC phosphorylation cascade in the kidneys of the mice receiving a normal diet. In the WNK4^+/^*^−^* mice, although the level of WNK4 protein expression in the kidneys was about half that of WNK4^+/+^ mice, levels of SPAK, OSR1 and NCC expression were not significantly altered by this degree of reduction in WNK4 expression. However, total NCC and NCC phosphorylated at S71 were almost completely absent in WNK4*^−^*^/^*^−^* mice (Figure 2C). In contrast with a previous report [13], a significant decrease in total and phosphorylated SPAK protein was evident in WNK4*^−^*^/^*^−^* mice, and total OSR1 was increased. Because WNK1 and WNK3 are known to phosphorylate OSR1/SPAK, we also examined their expression. WNK1 expression in the kidneys was significantly increased in WNK4*^−^*^/^*^−^* mice. Despite this, NCC phosphorylation remained almost absent. To clarify the segment of the kidney in WNK4*^−^*^/^*^−^* mice with increased WNK1 expression, we performed double immunofluorescence staining of WNK1 and total NCC. As shown in Supplementary Figure S1 (available at http://www.bioscirep.org/bsr/034/bsr034e107add.htm), the DCT were dilated, cell height was decreased and staining for NCC was reduced in the WNK4*^−^*^/^*^−^* mice. The WNK1 antibody used does not usually detect significant WNK1 expression in the kidneys of WNK4^+/+^ mice. However, increased WNK1 expression, co-localized with NCC expression, was evident in WNK4*^−^*^/^*^−^* mice, suggesting that a compensatory increase in WNK1 occurred in the DCT, but that this was insufficient to maintain NCC activity. This compensation might be caused by the increased transcription of the *WNK1* gene and/or by the reduced degradation of WNK1 protein. WNK3 protein expression was not detected by immunoblotting in the kidneys of either WNK4^+/+^ or WNK4*^−^*^/^*^−^* mice (results not shown), confirming that WNK3 is not a major WNK in the kidneys *in vivo* [28,29], even in the absence of WNK4.

We also investigated other transporters and channels thought to be regulated by WNK4. The levels of expression of both the full-length and cleaved forms of the α and γ subunits of ENaC were similar between WNK4^+/+^, WNK4^+/^*^−^* and WNK4*^−^*^/^*^−^* mice. Castañeda-Bueno et al. [13] did not present immunoblots for ENaC. However, they did perform functional assays to estimate ENaC activity, and showed that ENaC was activated to compensate for NCC inactivation resulting from WNK4 knockout. We thought that ENaC should be activated to compensate for NCC inactivation in our WNK4*^−^*^/^*^−^* mice, but the magnitude of difference may be insufficient for detection by immunoblotting, possibly because the phenotype of our WNK4*^−^*^/^*^−^* is milder than those analysed by Castañeda-Bueno et al. [13] given their serum potassium levels. However, ROMK was significantly increased in our WNK4*^−^*^/^*^−^* mice. NKCC2 expression and phosphorylation were not significantly affected by WNK4 knockout, consistent with our previous finding that the major WNK4 signal in the mouse kidney does not co-localize with NKCC2 [22].

### Involvement of WNK4 in low-potassium diet- and insulin-induced NCC activation

We previously identified regulators of the WNK–OSR1/SPAK–NCC signal cascade, including salt [11] and potassium intake [30], aldosterone [11], AngII [31,32] and insulin [20,33]. However, the identity of the WNK responsible for their regulation of NCC remains unclear. Low-salt diet- and AngII-induced NCC activation are reduced in WNK4 knockout mice [13]. Therefore, we investigated the involvement of WNK4 in the regulation of NCC by a low-potassium diet and insulin.

**Figure 3:**
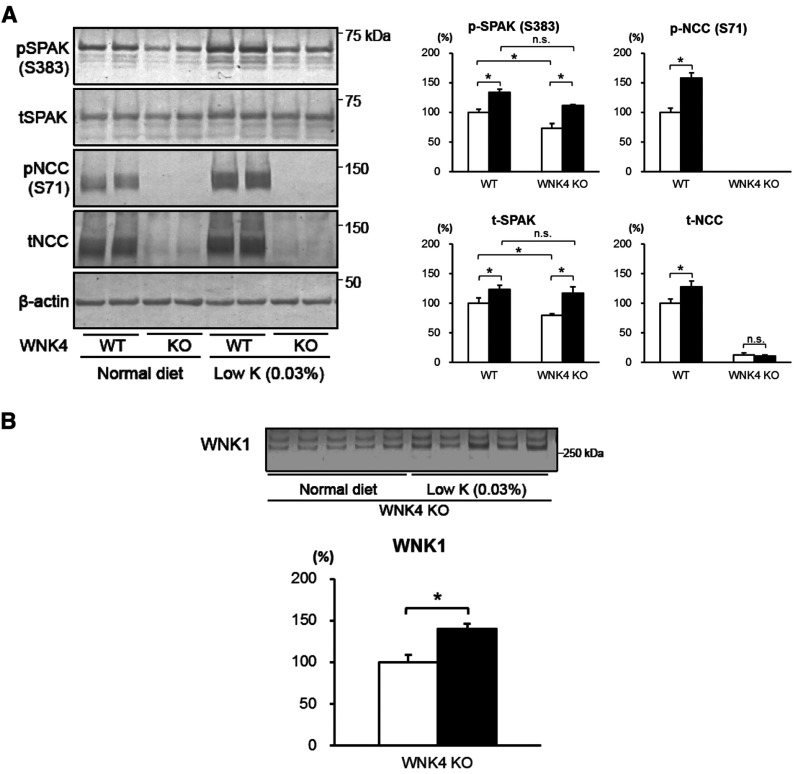
Responses to a low-potassium diet in WNK4*^−^*^/^*^−^* mice (**A**) Immunoblots of the kidney proteins of WNK4^+/+^ and WNK4*^−^*^/^*^−^* mice fed normal (open bars) or low-potassium (black bars) diets for 7 days before experiments (*P<0.05; *n*=5–6). Both WNK4^+/+^ and WNK4*^−^*^/^*^−^* mice showed increased phosphorylation of SPAK when fed the low-potassium diet. However, WNK4*^−^*^/^*^−^* mice did not exhibit increased phosphorylation of NCC. (**B**) In WNK4*^−^*^/^*^−^* mice, WNK1 increased with a low-potassium diet (black bar) compared with a normal diet (open bar; *P<0.05; *n*=5).

First, we fed the mice a low-potassium diet [0.03% (w/w) KCl] for 7 days. A low-potassium diet-induced increase in total and phosphorylated NCC observed in WNK4^+/+^ mice was not evident in WNK4*^−^*^/^*^−^* mice. In contrast, both WNK4^+/+^ and WNK4*^−^*^/^*^−^* mice exhibited increased SPAK phosphorylation, when fed a low-potassium diet (Figure 3A). We hypothesized that other WNKs may be involved in SPAK activation in nephron segments other than the DCT in WNK4*^−^*^/^*^−^* mice, and examined WNK1 expression in these mice. As expected, a further increase in WNK1 expression was evident in WNK4*^−^*^/^*^−^* mice fed a low-potassium diet (Figure 3B).

**Figure 4:**
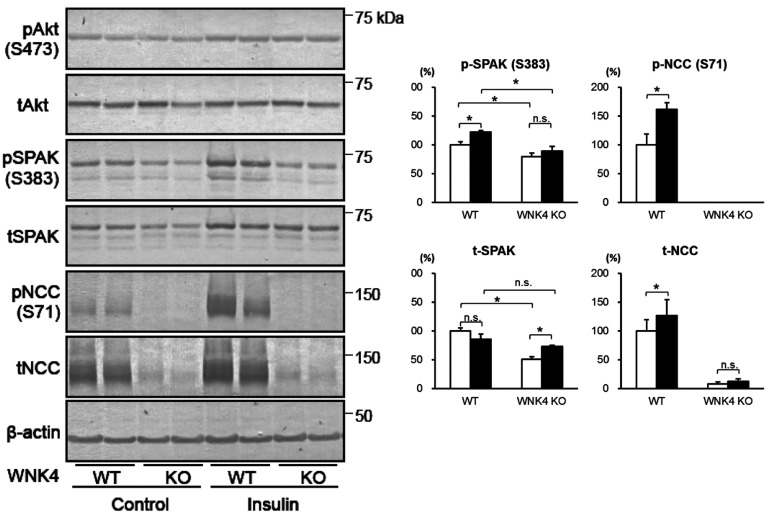
Response to acute insulin administration in WNK4*^−^*^/^*^−^* mice Immunoblots of kidney proteins from WNK4^+/+^ and WNK4*^−^*^/^*^−^* mice infused with vehicle (open bars) or insulin (black bars). Insulin-induced SPAK and NCC phosphorylation were not observed in WNK4*^−^*^/^*^−^* mice. Insulin was administrated intraperitoneally at a dose of 5 units/kg. Control mice received vehicle alone. Mice were killed 60 min after insulin injection (*P<0.05; *n*=5).

We then performed an acute insulin infusion experiment in WNK4*^−^*^/^*^−^* mice by administering insulin intraperitoneally at a dose of 5 U/kg, and killed the mice 60 min after injection. As shown in Figure 4, insulin-induced SPAK and NCC phosphorylation in the kidneys did not occur in WNK4*^−^*^/^*^−^* mice, indicating that the effect of insulin signalling on SPAK and NCC is exclusively mediated by WNK4.

### Role of WNK4 in the regulation of NCC in mice fed a high-salt diet

**Figure 5:**
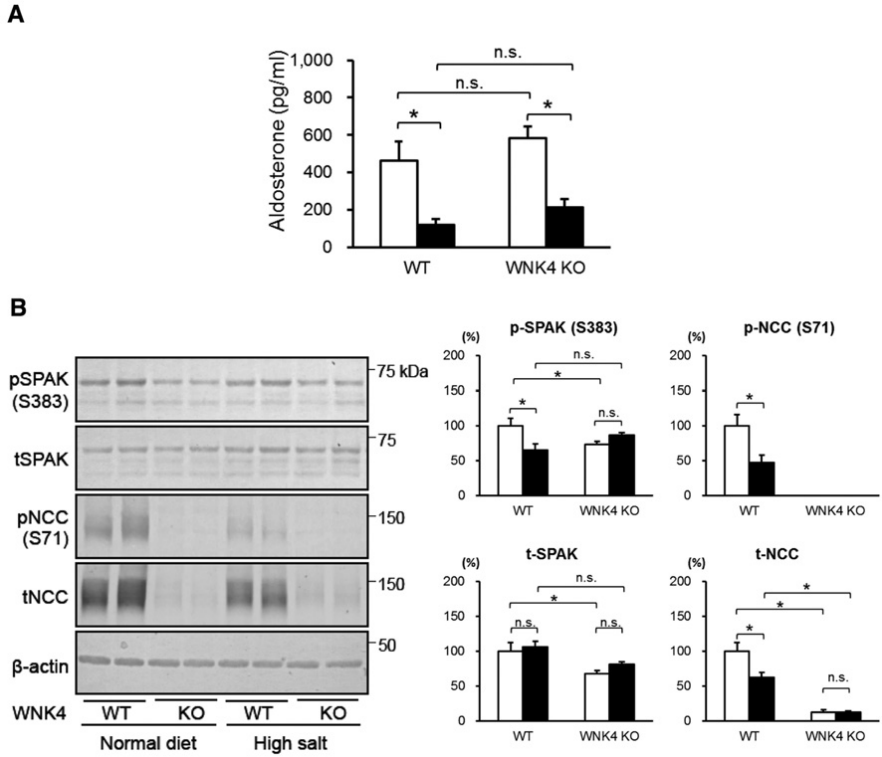
Response to a high-salt diet in WNK4*^−^*^/^*^−^* mice (**A**) Plasma aldosterone level in WNK4^+/+^ and WNK4*^−^*^/^*^−^* mice on a normal (open bars) or high-salt (black bars) diet. There was no difference between WNK4^+/+^ and WNK4*^−^*^/^*^−^* mice. A high-salt diet significantly decreased aldosterone levels in both groups (*P<0.05; *n*=5–6). (**B**) Immunoblots of the kidney proteins of WNK4^+/+^ and WNK4*^−^*^/^*^−^* mice fed a normal (open bars) or high-salt (black bars) diet for 7 days (*P<0.05; *n*=5). WNK4 knock out did not ameliorate the decreased phosphorylation and total amount of Na–Cl cotransporter caused by a high-salt diet.

We showed that WNK4 behaves as a positive regulator of NCC through the activation of SPAK and OSR1 in the kidneys *in vivo* [10,17]. However, some still believe that WNK4 is a negative regulator of NCC, switching to positive regulation only in the presence of AngII signalling [32]. Therefore, we investigated the role of WNK4 in NCC regulation under suppression of the RAA (renin-angiotensin-aldosterone) system affected by a high-salt diet. We fed the mice a normal [0.9% (w/w) NaCl] or high-sodium [4% (w/w) NaCl] diet for 7 days before the extraction of the kidney proteins and collection of blood samples. Aldosterone levels were significantly decreased by the high-salt diet in WNK4^+/+^ and WNK4*^−^*^/^*^−^* mice (Figure 5A), indicating that the RAA system was suppressed. As shown previously [11] and in Figure 5(B), a high-salt diet suppressed phosphorylation of SPAK and NCC and reduced total NCC in WNK4^+/+^ mice. If WNK4 is a negative regulator of NCC in the absence of AngII signalling in WNK4^+/+^ mice, NCC should be activated by a mechanism independent of WNK–OSR1/SPAK signalling in WNK4 knockout mice [8]. However, phosphorylated and total NCC did not increase in WNK4*^−^*^/^*^−^* mice compared with WNK4^+/+^ mice, remaining almost undetectable. Furthermore, BP in WNK4*^−^*^/^*^−^* mice did not increase compared with WNK4^+/+^ mice, even with a high-salt diet (Figure 2A), suggesting that NCC is never activated in the absence of WNK4. Thus, there is no evidence for a negative regulatory role of WNK4 in NCC regulation in the kidneys, even when RAA signalling is suppressed.

## DISCUSSION

In hypertension research, the mechanism of renal sodium transport regulation is a controversial issue. In this study, we clarified the essential role of WNK4 as a positive regulator of NCC in the kidneys using the WNK4 knockout mice. We demonstrated that NCC expression and phosphorylation are highly dependent on the presence of WNK4, and that the absence of WNK4 does not promote NCC expression under any of the conditions tested in this study.

There is now general consensus that the phosphorylation status of NCC reflects its *in vivo* activity [34]. NCC phosphorylation at specific N-terminal sites was shown to be important for its transport activity [34], and also for its localization at the apical plasma membrane [9,35]. We have often observed that a decrease in NCC phosphorylation is accompanied by a decrease in total NCC in the kidneys [10,11]. We believe that the primary change was in phosphorylation because we often observed the magnitude of change in phosphorylation was greater than that in total NCC. We recently showed that phosphorylation inhibits NCC ubiquitination, supporting this theory [36]. In this respect, NCC regulation by phosphorylation as a result of WNK–OSR1/SPAK signalling is the major mechanism of NCC regulation, and WNK4 must be considered an important positive regulator of NCC, as both WNK4 and WNK1 were shown to phosphorylate OSR1 and SPAK *in vitro* [12]. Furthermore, the discovery of new genes (*KLHL3* and *Cullin-3*) responsible for PHAII [14,15] supports the notion that WNK4 positively regulates NCC in the human kidney. Recently, we also generated several lines of wild-type WNK4–BAC TG mice, and showed that overexpression of WNK4 in the kidneys robustly increased SPAK and NCC phosphorylation and induced PHAII phenotypes [16]. Before this study, Lalioti et al. [37] reported that a single line of WNK4 TG mice appeared to show inhibition, rather than activation, of the NCC function. This data was the sole *in vivo* evidence validating the negative effect of wild-type WNK4 on NCC suggested by *in vitro* studies [5,6]. However, Lalioti et al. data [37] should be interpreted with caution, considering the pitfalls inherent in studies using TG mice [16].

In this study, WNK4*^−^*^/^*^−^* mice exhibited an almost complete absence of total and phosphorylated NCC on immunoblots, consistent with the data of Castañeda-Bueno et al. [13]. Although this group focused on the role of WNK4 in NCC regulation in the presence of AngII stimulation, their analyses of WNK4 knockout mice gave us further opportunity to investigate the role of WNK4 in NCC regulation under other conditions, thereby establishing a complete picture of the role of WNK4 in NCC regulation *in vivo*. As shown by our results, NCC was never up-regulated or activated by the deletion of WNK4 even in the absence of AngII stimulation. We are not denying the inhibitory effect of wild-type WNK4 on NCC observed in *Xenopus* oocytes [5,6] and cultured cells [38]. However, these inhibitory effects may be minimal in the kidneys *in vivo*, overwhelmed by the strong positive regulation of NCC by WNK4 via its phosphorylation. The importance of phosphorylation to the regulation of NCC is confirmed by the absence of PHAII phenotypes in NCC TG mice [39]. An increase in the abundance of NCC alone, without upstream WNK–OSR1/SPAK signalling, does not result in NCC activation.

We observed some minor phenotypic differences between our WNK4*^−^*^/^*^−^* mice and those used by Castañeda-Bueno et al. [13]. WNK4 knockout mice used by Castañeda-Bueno et al. [13] exhibited hypokalaemia, but our WNK4*^−^*^/^*^−^* mice did not exhibit hypokalaemia. We observed a slight but significant decrease in total and phosphorylated SPAK in our WNK4*^−^*^/^*^−^* mice, and an increase in total OSR1, but those used by Castañeda-Bueno et al. [13] did not exhibit any significant changes in SPAK and OSR1. The origin of these disparities is unclear, but differences in genetic background may provide one explanation, as both WNK4 knockout mice have a mixed background (C57BL6/J and 129Sv), and the contribution of these strains to each WNK4 knockout mouse may differ. Regarding serum potassium, even NCC knockout mice did not exhibit hypokalaemia [40], suggesting that normokalaemia in our WNK4*^−^*^/^*^−^* mice is not necessarily unexpected. It is also true that data on serum potassium in mice vary significantly depending on how blood samples are collected (after killing versus alive but under anaesthesia). As for the differences in SPAK and OSR1, these may originate from the antibodies used in the two studies.

We also obtained data on WNK-related molecules not described in the study by Castañeda-Bueno et al. [13]. We demonstrated that WNK1, but not WNK3, underwent a compensatory increase in WNK4*^−^*^/^*^−^* mice, further establishing the minor involvement of WNK3 in NCC regulation in the kidneys. However, this increase in WNK1 in the DCT may be insufficient to compensate for WNK4 deletion, as NCC phosphorylation was significantly decreased in WNK4*^−^*^/^*^−^* mice. Although insufficient to compensate for the absence of WNK4, this finding suggests that WNK1 has the same role as WNK4 in the DCT. As no controversy surrounds the positive influence of WNK1 on NCC, this data also supports the positive role of WNK4 in NCC regulation. An increase in WNK1 expression was also evident in WNK4*^−^*^/^*^−^* mice fed a low-potassium diet (Figure 3B). Increased WNK1 expression may explain the increase in SPAK phosphorylation, but did not contribute to NCC phosphorylation, suggesting that the increased phosphorylation of SPAK in WNK4*^−^*^/^*^−^* mice fed a low-potassium diet occurs in nephron segments other than the DCT.

For the first time, we have presented the status of ROMK in WNK4^−/−^ mice. Previously, ROMK was shown to be inhibited by wild-type WNK4, and strongly inhibited by PHAII-causing mutant WNK4 [41]. Through the analysis of *WNK*4*^D561A^*^/+^ PHAII model mice crossed with *Osr1* and *Spak* knock-in mice, we showed that the pathogenesis of hypokalaemia is also the result of NCC activation by WNK–OSR1/SPAK signalling, but is not caused by a direct effect of mutant WNK4 on ROMK. However, the effect of wild-type WNK4 on ROMK has not been clarified *in vivo*. We observed a clear increase in ROMK in the immunoblots of the total kidney samples from WNK4^−/−^ mice exhibiting normal serum potassium levels. Although further detailed analyses focusing on intracellular localization are necessary, our data suggest that wild-type WNK4 behaves as a negative regulator of ROMK in the kidneys, consistent with *in vitro* studies [41].

In conclusion, data obtained from our WNK4*^−^*^/^*^−^* mice under various conditions clearly show that WNK4 is a strong positive regulator, and never a negative regulator, of NCC in the mouse kidney *in vivo*.

## Online data

Supplementary data
