# Supplementary material for: WNK4 is the major WNK positively regulating NCC in the mouse kidney
Source: Biosci Rep. 2014 May 9;34(3):e00107. doi: 10.1042/BSR20140047 (PMC4212913; doi:10.1042/BSR20140047)
Supplement: Supplementary data [file bsr034e107add.pdf]

Biosci. Rep. (2014) / **34** / art:e00107 / doi 10.1042/BSR20140047

Original Paper

OPEN ACCESS

Bioscience Reports

www.bioscirep.org

Bioscience Reports

SUPPLEMENTARY DATA

WNK4 is the major WNK positively regulating NCC in the mouse kidney

Daiei TAKAHASHI*, Takayasu MORI*, Naohiro NOMURA*, Muhammad Zakir Hossain KHAN*, Yuya ARAKI*, Moko ZENIYA*, Eisei SOHARA*, Tatemitsu RAI*, Sei SASAKI* and Shinichi UCHIDA*[1]

*Department of Nephrology, Graduate School of Medical and Dental Sciences, Tokyo Medical and Dental University, 1-5-45 Yushima, Bunkyo, Tokyo 113-8519, Japan

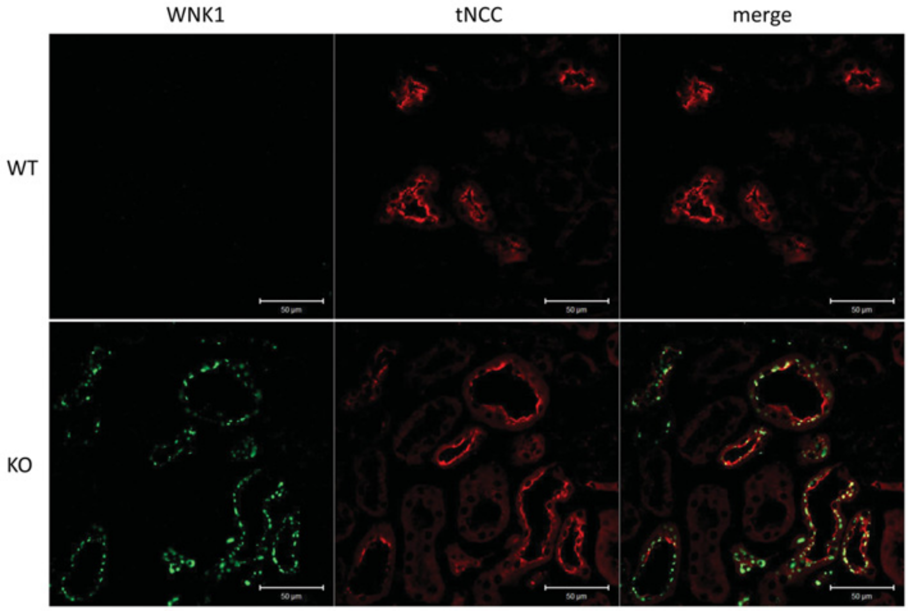

Figure S1 **Immunofluorescence of WNK1 and NCC in the kidney**
The immunofluorescence of WNK1 and NCC in the kidneys of WNK4$^{+/+}$ and WNK4$^{-/-}$ mice. An apparent increase in WNK1 in the kidneys of WNK4$^{-/-}$ mice was only observed in the distal convoluted tubules (DCT). However, the DCT appeared to be dilated with a reduced cell height and NCC immunofluorescence.

Received 6 March 2014/19 March 2014; accepted 21 March 2014

Published as Immediate Publication 21 March 2014, doi 10.1042/BSR20140047

[1] To whom correspondence should be addressed (email suchida.kid@tmd.ac.jp)

© 2014 The Author(s) This is an Open Access article distributed under the terms of the Creative Commons Attribution Licence (CC-BY) (http://creativecommons.org/licenses/by/3.0/) which permits unrestricted use, distribution and reproduction in any medium, provided the original work is properly cited.
